# Patterns of influenza B circulation in Latin America and the Caribbean, 2010–2017

**DOI:** 10.1371/journal.pone.0219595

**Published:** 2019-08-08

**Authors:** Rakhee Palekar, Angel Rodriguez, Cinthia Avila, Gisela Barrera, Miriam Barrera, Hebleen Brenes, Alfedo Bruno, Nathalie El Omeiri, Rodrigo Fasce, Walquiria Ferreira de Almeida, Danilo Franco, Maribel Huaringa, Jenny Lara, Roxana Loayza, Irma Lopez-Martinez, Terezinha Maria de Paiva, Jose Medina, Jenny Ojeda, Alba Maria Ropero, Viviana Sotomayor, Cynthia Vazquez, Marta Von Horoch

**Affiliations:** 1 Pan American Health Organization (PAHO/WHO), Washington D.C., United States of America; 2 Centro Nacional de Enfermedades Tropicales (CENETROP), Santa Cruz, Bolivia; 3 Instituto de Diagnóstico y Referencia Epidemiológicos (InDRE), Mexico City, México; 4 Laboratorio Nacional de Salud (LNS), Guatemala City, Guatemala; 5 Instituto Costarricense de Investigación y Enseñanza en Nutrición y Salud (INCIENSA), San Jose, Costa Rica; 6 Instituto Nacional de Investigación en Salud Pública (INSPI), Universidad Agraria del Ecuador, Guayaquil, Ecuador; 7 Instituto de Salud Pública de Chile, Santiago, Chile; 8 Ministerio de Salud, Brasilia, Brasil; 9 Instituto Conmemorativo Gorgas de Estudios de la Salud, Panama City, Panamá; 10 Instituto Nacional de Salud (INS), Lima, Perú; 11 Instituto Adolfo Lutz (IAL), Sao Paulo, Brasil; 12 Ministerio de Salud Pública, Quito, Ecuador; 13 Ministerio de Salud, Santiago, Chile; 14 Laboratorio Central de Salud Pública (LCSP), Asunción, Paraguay; 15 Ministerio de Salud Pública y Bienestar Social, Asunción, Paraguay; Columbia University, UNITED STATES

## Abstract

**Objective:**

There are limited published data about the circulation of influenza B/Victoria and B/Yamagata in Latin America and the Caribbean (LAC) and most countries have a vaccine policy that includes the use of the trivalent influenza vaccine. We analyzed influenza surveillance data to inform decision-making in LAC about prevention strategies, such as the use of the quadrivalent influenza vaccine.

**Methods:**

There are a total of 28 reference laboratories and National Influenza Centers in LAC that conduct influenza virologic surveillance according to global standards, and on a weekly basis upload their surveillance data to the open-access World Health Organization (WHO) platform FluNet. These data include the number of specimens tested for influenza and the number of specimens positive for influenza by type, subtype and lineage, all by the epidemiologic week of specimen collection. We invited these laboratories to provide additional epidemiologic data about the hospitalized influenza B cases. We conducted descriptive analyses of patterns of influenza circulation and characteristics of hospitalized cases. We compared the predominant B lineage each season to the lineage in the vaccine applied, to determine vaccine mismatch. A Chi-square and Wilcoxan statistic were used to assess the statistical significance of differences in proportions and medians at the *P*<0.05 level.

**Findings:**

During 2010–2017, the annual number of influenza B cases in LAC was ~4500 to 7000 cases. Since 2011, among the LAC-laboratories reporting influenza B lineage using molecular methods, both B/Victoria and B/Yamagata were detected annually. Among the hospitalized influenza B cases, there were statistically significant differences observed between B/Victoria and B/Yamagata cases when comparing age and the proportion with underlying co-morbid conditions and with history of oseltamivir treatment (*P*<0.001). The proportion deceased among B/Victoria and B/Yamagata hospitalized cases did not differ significantly. When comparing the predominant influenza B lineage detected, as part of surveillance activities during 63 seasons among 19 countries, to the lineage of the influenza B virus included in the trivalent influenza vaccine used during that season, there was a vaccine mismatch noted during 32% of the seasons analyzed.

**Conclusions:**

Influenza B is important in LAC with both B/Victoria and B/Yamagata circulating annually in all sub regions. During approximately one-third of the seasons, an influenza B vaccine mismatch was identified. Further analyses are needed to better characterize the medical and economic burden of each influenza B lineage, to examine the potential cross-protection of one vaccine lineage against the other circulating virus lineage, and to determine the potential impact and cost-effectiveness of using the quadrivalent vaccine rather than the trivalent influenza vaccine.

## Background

Seasonal influenza viruses circulate annually and cause disease in humans. In temperate countries, there is typically one influenza season annually, while in tropical countries, the seasonality of influenza can vary with typically two periods of peak virus activity annually. There are four groups or types of seasonal influenza viruses—influenza A, B, C and D. Type C influenza viruses cause mild human infection and are associated with sporadic cases and as such, active surveillance is not conducted for influenza C viruses. Influenza D viruses primarily affect cattle and are not known to infect or cause illness in people [1]. Among influenza A and B viruses, influenza A viruses tend to predominate and during each season, a variable percent of influenza A viruses are of the H1 subtype and of the H3 subtype. While influenza A viruses predominate during influenza seasons compared to influenza B viruses, influenza B viruses are detected globally each season in both tropical and temperate countries [2,3].

Currently, there are two distinct influenza B lineages circulating globally—B/Victoria and B/Yamagata [4–7]. The B/Victoria lineage gradually emerged from China, in 1975 and was the dominant influenza B lineage detected globally in the late 1980s; while the B/Yamagata lineage appeared in Japan in 1990 and during the next 10 years, was the dominant influenza B lineage circulating globally[4–7]. Since the 2001–2002 influenza season, both of these lineages have been circulating and in fact during many influenza seasons, both lineages circulate in equal proportions [4–7].

Vaccination is the most effective way to prevent influenza infection. Since 2010, there have been two compositions of influenza vaccine available each season—a trivalent vaccine and a quadrivalent vaccine[8]. The trivalent vaccines protect against three different influenza viruses—two influenza A viruses and one lineage of influenza B virus; the quadrivalent vaccines protect against four different influenza viruses—two influenza A viruses and a B/Victoria and a B/Yamagata influenza virus. In Latin America and the Caribbean (LAC) there are 38 out of 50 countries and territories that have a vaccine policy for the annual use of seasonal influenza vaccine[9]. As of 2017, 90% of these countries and territories were using the trivalent influenza vaccine formulation, which is less expensive that the quadrivalent influenza vaccine (~$1 USD per dose versus $5 USD per dose, respectively) [10]. Among countries with data available, the median vaccination coverage during 2017 was 59% (range: 1–97) among children and 65% (range: 2–100) among the elderly [11].

To date, there are limited published data about the circulation of influenza B viruses in LAC, which are essential to understand their epidemiologic patterns and the potential relevance of using a quadrivalent vaccine. In order to answer these questions, we analyzed patterns of influenza B circulation in LAC, compared the epidemiologic characteristics of hospitalized influenza B/Victoria to influenza B/Yamagata cases, and assessed the concordance between the predominant influenza B lineage and the B lineage included in the vaccine applied in each country.

## Methods

The Global Influenza Surveillance and Response System (GISRS) is a network that as of 2017, included 141 National Influenza Centers (NICs), six Collaborating Centers (CCs), and four Essential Regulatory Laboratories that conduct influenza virologic surveillance according to terms of reference established by the World Health Organization (WHO)[12]. As of 2017 in LAC, there were a total of 24 NICs and an additional four laboratories [13] that conducted influenza surveillance according to these global standards, covering a total of 34 LAC countries and territories. These laboratories receive samples from sentinel sites conducting influenza surveillance, primarily in hospitals, but also in some ambulatory primary-care settings. They also receive samples collected upon clinicians’ request for diagnostic testing. Laboratories use a combination of indirect immunofluorescence and real-time polymerase chain reaction (rRT-PCR) to test for the presence of influenza viruses. Real-time RT-PCR testing is conducted using molecular detection kits provided by the WHO CC for Influenza Surveillance at the U.S. Centers for Disease Control and Prevention (CDC) [13,14]. Influenza A viruses are further subtyped, and influenza B viruses are tested for lineage, by rRT-PCR. The WHO CC at the U.S. CDC began to provide influenza B lineage detection molecular kits in 2013 to countries in LAC free-of-charge. The percent of the population covered by the surveillance in each country has not been systematically documented, but in most of the countries, samples are collected from all parts of the country for testing at the NIC.

All LAC laboratories that conduct surveillance according to the global WHO standards, report the total number of samples tested for influenza and the number positive by type, subtype and lineage to the GISRS network’s open-access online platform FluNet through the Pan American Health Organization (PAHO)/WHO. We extracted the number of positive influenza samples by type, subtype and lineage, as well as the total number of samples tested for influenza by epidemiologic week of sample collection from FluNet for these LAC-laboratories (n = 28) for the period from 2010–2017. We then invited all LAC-laboratories that participate in the GISRS network (n = 28) to provide epidemiologic information about the hospitalized influenza B cases—hospitalization status, history of treatment with oseltamivir, history of receipt of the current influenza vaccine, and final clinical outcome.

We conducted a descriptive analysis of the data comparing the case counts of and the timing of the circulation of influenza A versus B as well as the hospitalization status, age, presence of co-morbidities, history of vaccine receipt, treatment with oseltamivir, in-hospital death, and the final clinical outcome at the time of discharge for influenza B/Victoria versus B/Yamagata cases. Co-morbidities were defined based upon the WHO list of pre-existing medical illness or co-morbid conditions (e.g. chronic respiratory disease, asthma, diabetes, chronic cardiac disease, chronic liver disease, chronic renal disease, chronic neurological or neuromuscular disease, hematological disorders, immunodeficiency) [15]. We used Chi-square and Wilcoxan statistics to assess the statistical significance of differences between proportions and medians at the *P*<0.05 level. Additionally, for each influenza season, among countries that had lineage information for at least ten influenza B cases, we also determined the B lineage that predominated each season. A lineage was considered predominant if it represented more than 60% of all influenza B cases that season; the lineages were considered co-dominant if either lineage represented 40 to 60% of all influenza B cases. Seasons that had a predominance of the B lineage that was different from the lineage included in the trivalent vaccine and seasons that had co-dominance of the B lineages, were considered vaccine mismatched seasons.

All analyses were conducted using Stata version 9.0. All data were considered exempt from ethical review, as they were de-identified and captured as part of routine surveillance activities.

## Results

**Fig 1:**
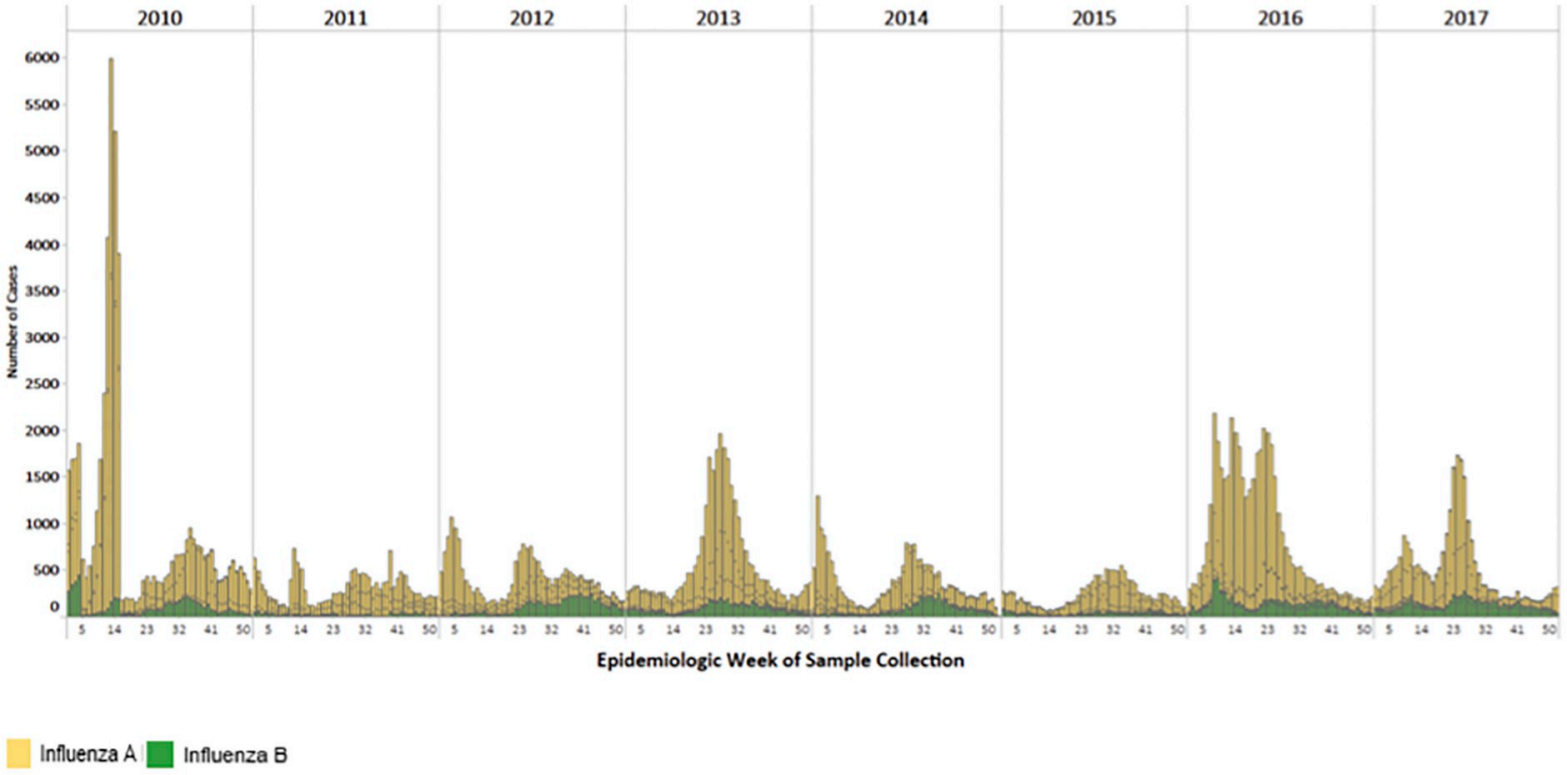
Distribution of Influenza Cases in Latin America and the Caribbean, 2010–2017. *Latin America and the Caribbean: Anguilla,Argentina, Aruba, Barbados, Belize, Bermuda, Bolivia (Plurinational State of), Brazil, Cayman Islands, CARPHA, Costa Rica, Colombia, Chile, Dominica, Dominican Republic, Ecuador, El Salvador, French, Guiana, Guatemala, Haiti, Honduras, Jamaica, Martinique, Mexico, Nicaragua, Panama, Paraguay, Peru, Saint Lucia, Suriname, Trinidad and Tobago, Uruguay and Venezuela (Bolivarian Republic of).

**Fig 2:**
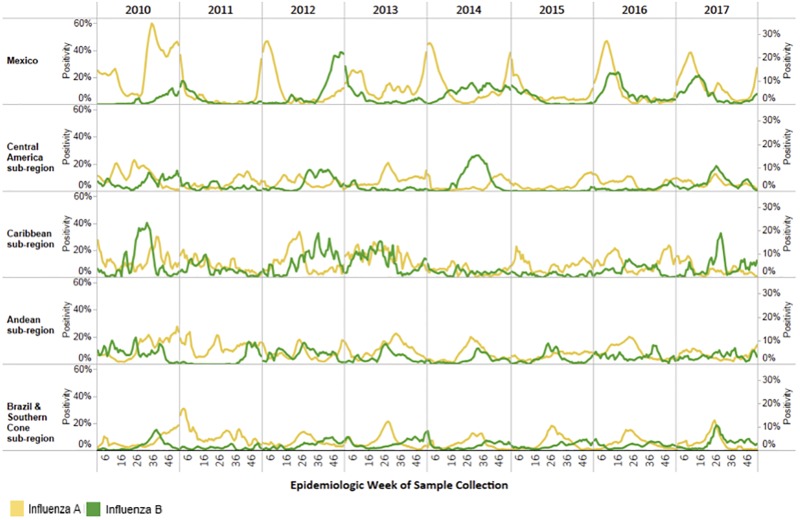
Percent Positivity for Influenza A and B in Latin America and the Caribbean, 2010–2017. *Central American sub-region: Costa Rica, El Salvador, Guatemala, Honduras, Nicaragua, and Panama Caribbean sub-region:: Anguilla, Aruba, Barbados, Belize, Bermuda, CARPHA, Cayman Islands, Dominica, Dominican Republic, French Guiana, Haiti, Jamaica, Martinique, Saint Lucia, Suriname and Trinidad and Tobago. Andean sub-region: Bolivia (Plurinational State of), Colombia, Ecuador, Peru, and Venezuela (Bolivarian Republic of) Southern Cone sub-region: Argentina, Brazil, Chile, Paraguay, and Uruguay.

During 2010–2017, 28 LAC laboratories detected n = 182,813 influenza A and 38,456 influenza B cases (Fig 1). While influenza A cases predominated during the period of analysis, compared to influenza B, there were a total of 4500–7000 influenza B cases annually (Table 1). Influenza B cases were detected in all geographic subregions of LAC (Fig 2). Among all influenza cases detected, the percentage of influenza samples in a given year that were reported to be influenza B varied from 1% to 54% (Table 1). There was year-to-year variability in all sub-regions (S1 Table). The timing of the circulation of influenza B varied by year and subregion (Fig 2). In the tropical Andean and Central American subregions, influenza B was detected year-round while in the temperate Southern Cone, influenza B was mostly detected at the end of the influenza season (Fig 2).

**Table 1:** Relative proportion of influenza B cases in Latin America and the Caribbean*, 2010–2017.

		2010		2011		2012		2013		2014		2015		2016		2017	
		n	%	n	%	n	%	n	%	n	%	n	%	n	%	n	%
**Mexico**†	Influenza B	485	16	130	2	1017	36	468	6	770	31	1,950	21	1305	24		
	Total influenza cases	3,078		5,686		2,835		7,342		2,524		9,172		5479			
**Central American sub-region**	Influenza B	1,310	5	218	13	960	44	152	6	1,093	54	10	1	217	10	905	41
	Total influenza cases	25,983		1,624		2,169		2,369		2,017		938		2,115		2,193	
**Caribbean sub-region**	Influenza B	212	11	168	21	547	43	429	22	233	36	72	6	756	32	261	41
	Total influenza cases	1,935		807		1268		1,927		652		1,228		2,350		637	
**Andean sub-region**	Influenza B	622	16	395	6	1,114	26	907	15	551	20	285	18	703	16	634	23
	Total influenza cases	3,977		6,988		4,334		6,227		2,775		1,567		4,486		2,816	
**Brazil & Southern Cone sub-region**	Influenza B	3,329	26	396	6	2,270	28	3,108	19	1,528	21	1,519	20	3,715	14	3,712	24
	Total influenza cases	12,938		6,436		8,181		16,473		7,204		7,581		27,517		15,441	

*Central American sub-region: Costa Rica, El Salvador, Guatemala, Honduras, Nicaragua, and Panama

Caribbean sub-region: Anguilla, Aruba, Barbados, Belize, Bermuda, CARPHA, Cayman Islands, Dominica, Dominican Republic, French Guiana, Haiti, Jamaica, Martinique, Saint Lucia, Suriname and Trinidad and Tobago.

Andean sub-region: Bolivia (Plurinational State of), Colombia, Ecuador, Peru, and Venezuela (Bolivarian Republic of)

Southern Cone sub-region: Argentina, Brazil, Chile, Paraguay, and Uruguay

^†^Period of analysis for Mexico is October to September

**Fig 3:**
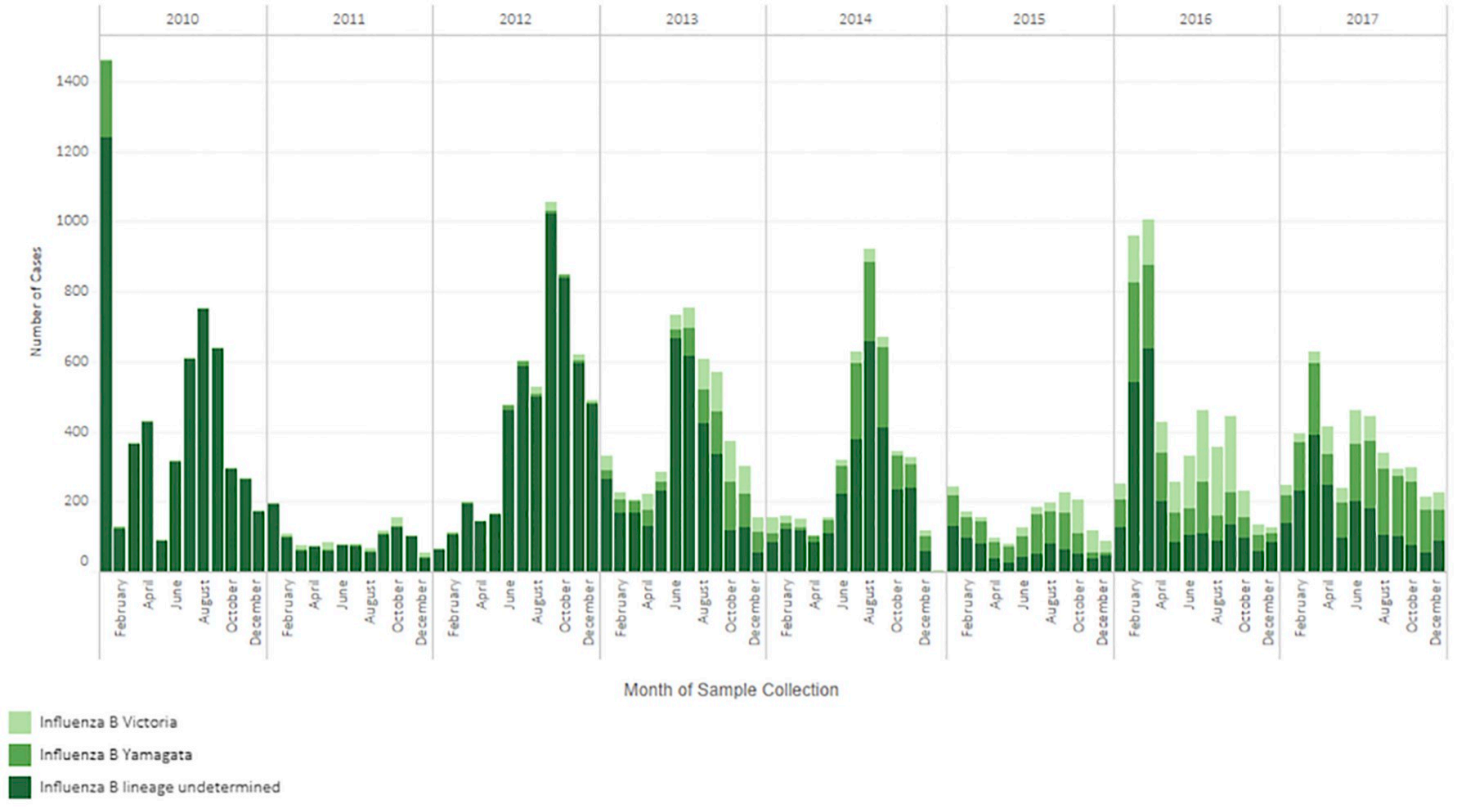
Distribution of influenza B cases among selected Countries in Latin America and the Caribbean, 2010–2017. *Data source Flu Net: Anguilla, Aruba, Barbados, Belize, Bermuda, Cayman Islands, Chile, Cuba, CARPHA, Dominica, Dominican Republic, Ecuador, French Guiana, Guatemala, Haiti, Honduras, Jamaica, Martinique, Nicaragua, Panama, Peru, Saint Lucia, Suriname, Trinidad and Tobago, Uruguay, Venezuela; Data source FluNet plus additional lineage data: Argentina, Brazil (states of Distrito Federal, Goias, Mato Grosso, Mato Grosso do Sui, Piaui, Rondonia, Sao Paulo, Tocantins), Colombia, Costa Rica, El Salvador, Mexico, Paraguay.

**Fig 4:**
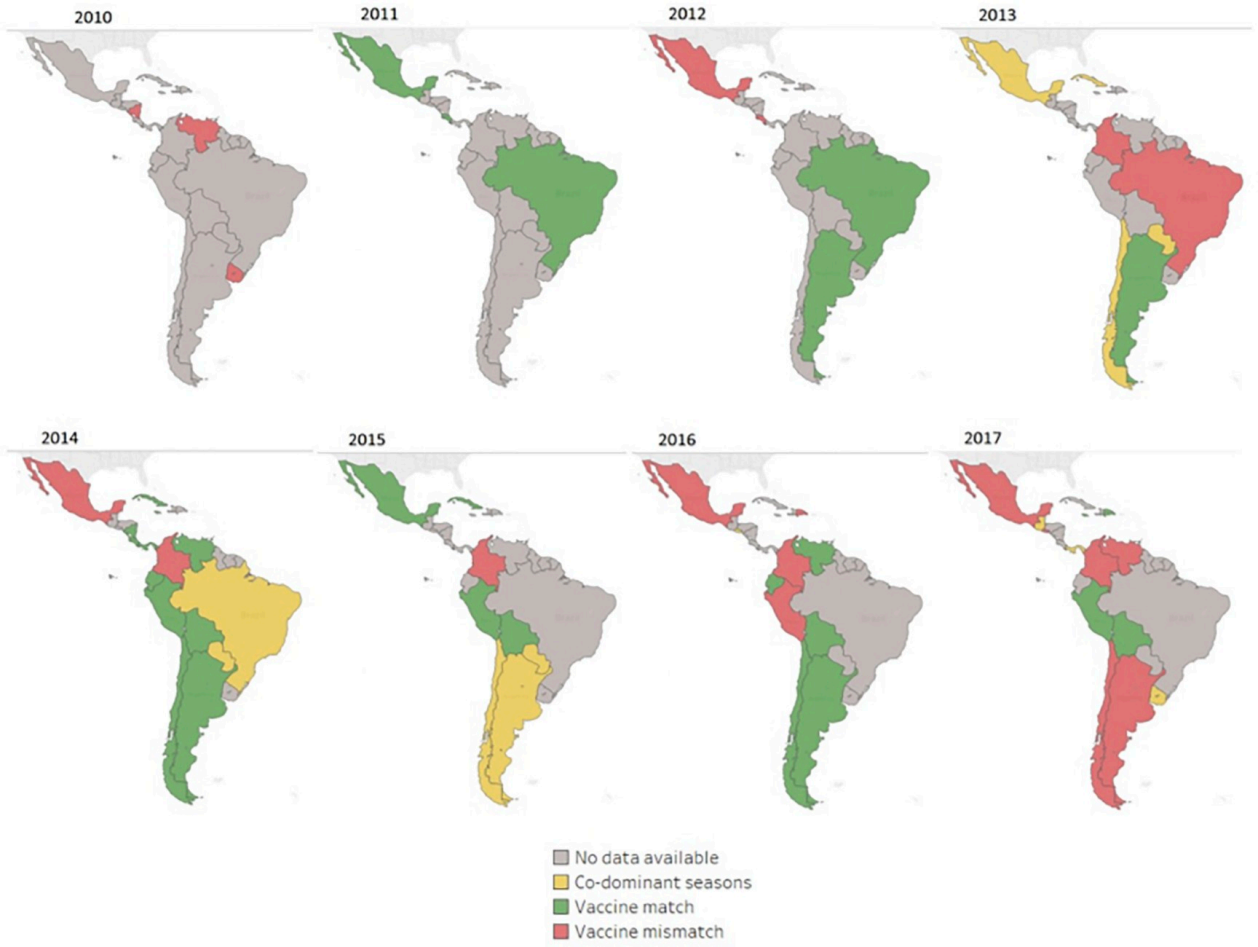
Comparison of influenza B lineage predominance and B lineage included in trivalent influenza vaccine used—Latin America and the Caribbean, 2010–2017. *Data from Brazil are from the states of: Distrito Federal, Goias, Mato Grosso, Mato Grosso do Sui, Piaui, Rondonia, Sao Paulo, Tocantins.

Since 2011, 26 LAC laboratories reporting influenza B lineage data to FluNet using molecular methods, detected both B/Victoria and B/Yamagata annually (Fig 3). In 2013, almost equal numbers of B/Victoria and B/Yamagata viruses were detected (Fig 3); however, in 2014 and 2015, there was a predominance of B/Yamagata over B/Victoria. When these lineage data were further disaggregated by country there was variability by country and year in terms of predominance of B/Victoria or B/Yamagata lineages (Table 2). When restricting the analysis to those seasons with at least 10 influenza B viruses with lineage information (n = 63), there was a predominant lineage during 79% (n = 50) of seasons and co-dominance during 21% (n = 13) of seasons. When comparing the predominant influenza B lineage detected as part of virologic surveillance, to the lineage of the influenza B virus included in the trivalent influenza vaccine, there was a mismatch during 32% (n = 20) of seasons (Fig 4) and when co-dominant seasons were considered as mismatched seasons as well, the number of mismatched seasons increased to 52% (n = 33) of seasons.

**Table 2:** Distribution of influenza B/Victoria and B/Yamagata cases among selected countries Latin America and the Caribbean*, 2010–2017.

Country	B/Victoria n (%)	B/Yamagata n (%)	Lineage undetermined n (%)	Total Influenza B Cases (100%)
Argentina	330 (6)	477 (9)	4409 (85)	5216
Brazil	297 (4)	399 (5)	7744 (91)	8440
CARPHA	28 (4)	24 (4)	596 (92)	648
Chile	1115 (26)	1537 (36)	1590 (37)	4242
Colombia	94 (16)	62 (11)	429 (73)	585
Costa Rica			864 (100)	864
Cuba	66 (8)	190 (23)	577 (69)	833
Dominican Republic	102 (21)	247 (50)	144 (29)	493
Ecuador	32 (5)	16 (2)	605 (93)	653
El Salvador	8 (2)	57 (13)	374 (85)	439
French Guiana	85 (23)	225 (60)	63 (17)	373
Guatemala	12 (1)	13 (1)	970 (98)	995
Haiti	11 (15)	50 (67)	14 (19)	75
Honduras			401 (100)	401
Jamaica	45 (21)	27 (13)	138 (66)	210
Martinique		17 (40)	26 (60)	43
Mexico	104 (2)	361 (5)	6441 (93)	6906
Nicaragua		41 (2)	1675 (98)	1716
Panama	91 (20)	111 (25)	248 (55)	450
Paraguay	56 (3)	76 (5)	1550 (92)	1682
Peru	101 (8)	459 (36)	714 (56)	1274
Uruguay	102 (6)	249 (14)	1405 (80)	1756
Venezuela	24 (15)	89 (55)	49 (30)	162
**Total**	**2703 (7)**	**4727 (12)**	**31026 (81)**	**38456**

*Caribbean Public Health Agency (CARPHA) data are from the following countries and territories: Anguilla, Aruba, Barbados, Belize, Bermuda, Cayman Islands, Dominica, Saint Lucia, Suriname and Trinidad and Tobago

Among countries (n = 14) providing epidemiologic data about hospitalized influenza B cases (n = 2,354), the median age and the percent deceased in the hospital among B/Victoria cases and B/Yamagata cases, did not differ significantly (Table 3). Persons hospitalized with B/Yamagata were more likely to have at least one underlying co-morbidity (*P*<0.001), have received the current influenza vaccine (*P* = 0.015), and have received antiviral treatment (*P*<0.001), compared to persons hospitalized with B/Victoria (Table 3).

**Table 3:** Comparison of influenza B/Victoria and B/Yamagata hospitalized cases among selected countries Latin America and the Caribbean*, 2010–2017.

	Influenza B Hospitalized Cases (n = 2,354)		
	B/Victoria n = 837	B/Yamagata n = 1,517	*P value*
Median age in years (Min-Max)	20 (0–97)	37 (0–94)	<0.001^§^
Treatment with oseltamivir % (n)	16.2 (n = 136)	29.7 (n = 451)	<0.0001
Co-morbid conditions†% (n)	7.9 (n = 66)	16.2 (n = 246)	<0.0001
Vaccination against influenza during the current season % (n)	7.8 (n = 65)	10.9 (n = 165)	0.0150
Deaths (in-hospital) % (n)	1.9 (n = 16)	3.3 (n = 50)	0.0515

* Argentina, Bolivia, Brazil (states of Distrito Federal, Goias, Mato Grosso, Mato Grosso do Sul, Piaui, Rondonia, Sao Paulo, Tocantins), Colombia, Costa Rica, Chile, El Salvador, Ecuador, Guatemala, Mexico, Panama, Paraguay, Peru, Uruguay.

^†^Defined as at least one comorbid condition

^§^Wilcox test

## Discussion

This analysis of the patterns of influenza B circulation in Latin American and the Caribbean provides important information to guide influenza prevention and control strategies. First, influenza B viruses represented 11–26% of all influenza cases in LAC annually. In some sub-regions, and during certain years, they represented as much as 43% of all influenza cases. These findings are similar to those of studies from other regions, reporting influenza B cases to represent 20–50% of influenza cases detected during a season ([3,4]. These results suggest that influenza B is an important contributor to influenza disease. Countries estimating influenza burden should contemplate estimates by type of influenza virus. Second, while influenza B has often been described as circulating at the end of the influenza season in temperate countries, we found, especially in tropical countries, that influenza B circulates year-round. This pattern of year-round circulation in tropical countries has been described in other regions globally [3,7] and as such, typing of influenza viruses, as part of virologic surveillance, should be conducted throughout the year [16].

Next, both influenza B lineages circulated annually in LAC, during our study period. There was no clear pattern to this circulation and countries within the same geographic sub region often had differing lineage predominance. Additionally, mismatch between the trivalent vaccine lineage and the predominant lineage occurred in approximately one third of the seasons analyzed and when considering the seasons with co-circulation this mismatch was more than 50%. These observations are important considering the exclusive-use of the trivalent vaccine in LAC as of 2017. Studies in Latin American countries using the trivalent vaccine have shown vaccine effectiveness (VE) against hospitalized influenza B to be 34% (95% confidence limit: -4; 58%) [17], but these studies did not estimate VE by lineage of influenza B. Other observational studies to date have shown some cross-protection between lineages in seasons with influenza B lineage mismatch [17–19] and considering the current market prices of quadrivalent vaccines, which represent a significant expenditure for immunization programs, careful consideration will need to be given to the cost-effectiveness of the use of a quadrivalent vaccine in LAC [20].

Finally, when comparing the characteristics of persons hospitalized with influenza B/Victoria to those hospitalized with B/Yamagata, cases of B/Yamagata were older, more likely to have an underlying co-morbid condition, have received the current influenza vaccine, and have been treated with oseltamivir [21]. The older age of the B/Yamagata cases could explain the increased co-morbidities and hence the higher likelihood of treatment with oseltamivir; this pattern of older age among B/Yamagata cases as compared to B/Victoria cases has been shown previously [22] Additionally, one important factor that we did not examine was the clinical severity of influenza B cases that might have been higher with B/Yamagata cases and hence might explain the higher likelihood for treatment with oseltamivir. However, as the proportion of deaths did not differ between the B/Victoria and B/Yamagata cases, it is less likely that the clinical severity differed between the two groups. The higher likelihood of vaccination among B/Yamagata cases, potentially implying lower vaccine effectiveness in this group is one that is best examined as part of a dedicated influenza vaccine effectiveness analysis, as mentioned above.

There are three key limitations to this analysis. First, not all countries in LAC determined the lineage of their influenza B cases and as such this analysis represents a subset of influenza B cases in LAC. Second, among the countries determining the lineage of their influenza B cases, some are not doing this lineage determination for all of their influenza B cases and as such, lineage data might be biased if cases are not being selected randomly for lineage determination. Finally, as the percent of the population covered by the surveillance is unknown in each country, this analysis cannot necessarily be generalized to the whole country.

In conclusion, influenza B contributes substantially to the annual number of influenza cases in LAC, with both B/Victoria and B/Yamagata viruses circulating in all sub-regions each year. Influenza B vaccine-virus mismatch among countries using the trivalent influenza vaccine has occurred but its effect hasn’t been assessed. Countries should continue to strengthen their influenza surveillance systems in order to identify and characterize influenza B cases, including through systematic lineage determination. Further analyses are needed to determine the medical and economic burden of influenza B/Victoria and B/Yamagata viruses, to examine the cross-protection between mismatched (vaccine virus versus predominant circulating virus) influenza B lineages, and to determine the potential impact and cost-effectiveness of using a quadrivalent influenza vaccine.

## Supporting information

S1 Table Relative proportion of influenza B cases in Latin America and the Caribbean, 2010–2017. (TIFF)
