# Supplementary figures and images for: Patterns of influenza B circulation in Latin America and the Caribbean, 2010–2017
Source: PLoS One. 2019 Aug 8;14(8):e0219595. doi: 10.1371/journal.pone.0219595 (PMC6687279; doi:10.1371/journal.pone.0219595)

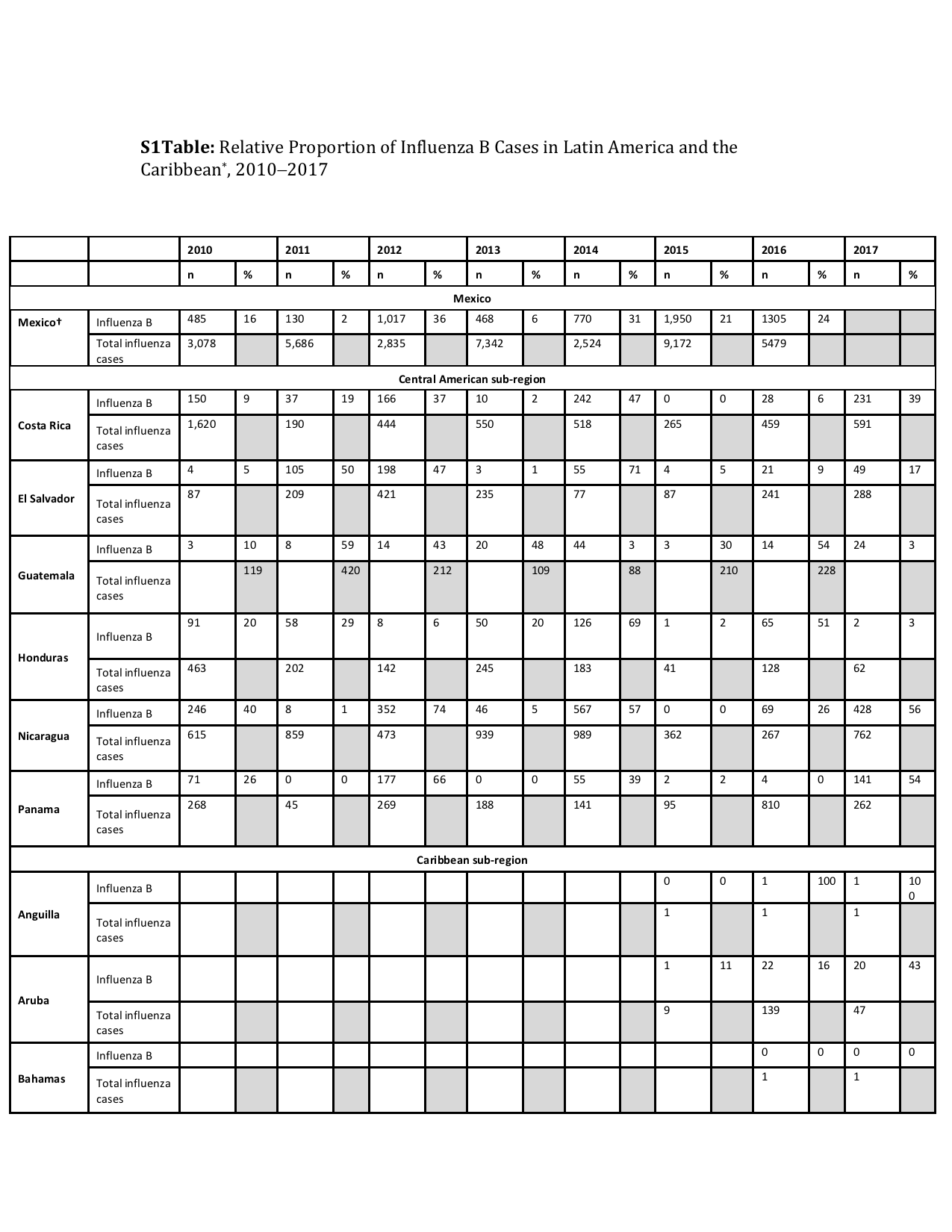

Supplement: S1 Table — (TIFF) [file pone.0219595.s001.tiff]
